# EXPANDING THE NETWORK: NONTRADITIONAL ANIMAL MODELS IN GEROSCIENCE

**DOI:** 10.1093/geroni/igz038.024

**Published:** 2019-11-08

**Authors:** Alan A Cohen

**Affiliations:** University of Sherbrooke, Sherbrooke, Quebec, Canada

## Abstract

Important insights into the biology of aging are coming from research on animals that have not been traditional staples of geroscience research. This symposium will highlight cutting-edge approaches to understand aging biology in the African killifish, exceptionally long-lived mammals such as the Naked Mole Rat, and across primate species, including the short-lived Marmoset.

